# Prevalence of Keratoconus in the Young Eastern Population of Saudi Arabia

**DOI:** 10.7759/cureus.55692

**Published:** 2024-03-06

**Authors:** Ashbala Khattak, Abid Altalhi, Anwar B Alotaibi, Aadam M Khattak

**Affiliations:** 1 Ophthalmology, Johns Hopkins Aramco Healthcare, Dhahran, SAU; 2 Epidemiology and Public Health, Johns Hopkins Aramco Healthcare, Dhahran, SAU; 3 Medicine, Weill Cornell Medicine, Doha, QAT

**Keywords:** progressive vision loss, crosslinking, early screening, young population, keratoconus (kc)

## Abstract

Background: Keratoconus (KCN) is a progressive corneal ectasia that manifests at a young age and significantly impacts vision and quality of life. Early diagnosis allows for effective treatment with corneal collagen crosslinking, yet there is a lack of screening methods. This research aims to screen adolescents and young adults for this sight-threatening disease using quick corneal tomography mapping.

Methods: This prospective cross-sectional study is being conducted at Johns Hopkins Aramco Healthcare in Saudi Arabia, focusing on subjects aged 13-23. We are presenting the data from our study as internal pilot study data. Bilateral corneal imaging with Pentacam HR (Oculus, Wetzlar, Germany), utilizing Scheimpflug corneal tomography, was performed. Historical data on allergies, eye rubbing, KCN, family history, previous eye surgery, and contact lens use were collected. The Belin Ambrosio Enhanced Ectasia Display total D value served as an objective criterion for suspect KCN (SKCN) diagnosis.

Results: In this study with 110 participants, KCN was identified in 2.75% of participants and SKCN in 11.93%. Systemic allergies or eczema were reported by 2.80%, with no cases in the KCN or SKCN groups. Eye rubbing behavior was observed in 5.50%, with the highest prevalence (33.30%) in the KCN group. A family history of KCN was found in 21.10%, with SKCN having the highest prevalence (30.80%).

Conclusion: This restricted population study reveals a significant KCN rate of 2.75%. The condition, easily detected and treatable with corneal collagen crosslinking, highlights the need for larger population studies to determine the disease's true prevalence. Efficient screening programs tailored to regional data are essential for early detection and intervention.

## Introduction

Keratoconus (KCN) is a progressive corneal ectasia characterized by corneal thinning, weakening, and forward protrusion, causing irregular astigmatism that leads to poor vision [1]. The disease is bilateral and typically exhibits asymmetric progression, often resulting in a delayed diagnosis. Although the etiology of KCN remains uncertain, factors such as genetic makeup, systemic allergies, and eye rubbing contribute to its manifestation [2].

A study conducted in the USA reported a prevalence of 0.05% in 1986 [3]. Subsequent studies, particularly in the Middle Eastern region, revealed a higher incidence of the disease [4-7] The harsh desert environment, with a high prevalence of allergic eye conditions and consanguinity (suggesting a potential genetic etiology), could be the factors responsible for the higher prevalence of KCN in the Middle Eastern region.

Unfortunately, KCN tends to manifest when vision has significantly deteriorated. Early diagnosis is crucial, given the treatable nature of the condition. In 2003, a novel treatment known as corneal collagen crosslinking was introduced, effectively strengthening the cornea and stabilizing the disease [8]. This treatment has globally reduced the need for corneal transplantation, as recent data indicates [9-10]. Early diagnosis, especially with the availability of cross-linking treatment, is critical to preventing further vision impairment.

The objective of our study is to identify the prevalence of KCN and design larger screening studies based on the data obtained in this research.

## Materials and methods

This prospective observational single-center study is underway at the Johns Hopkins Aramco Healthcare (JHAH) facility in the eastern province of Saudi Arabia. It constitutes a pilot study within a four-year research initiative aimed at determining the prevalence of KCN in this region. Subjects between 13 and 23 years old were recruited from the hospital's patient population through advertising on electronic media portals, mass emails, and physical displays of study information throughout the hospital. Subjects with a history of KCN, refractive surgery, any other corneal surgery, or any contact lens use except for occasional use were excluded from participation.

All subjects, or their parents, if below 18, provided informed consent. Detailed information regarding a family history of KCN, systemic allergies, eye rubbing, or other systemic syndromes was collected. Subjects with occasional contact lenses confirmed no contact lens use at least two weeks prior to corneal imaging. Bilateral corneal imaging was performed using Pentacam HR (Oculus, Wetzlar, Germany), a rotating Scheimpflug anterior segment tomography device that captures three-dimensional images. Images were taken by an experienced technician, ensuring quality specifications were met, with only those labeled "OK" being utilized. An experienced corneal surgeon assessed the images, employing objective criteria such as keratometry values, maximum keratometry, thinnest corneal pachymetry, and Belin Ambrosio Enhanced Ectasia Display total D values to identify KCN patients or suspects. Those with a D value exceeding 1.6 but less than 2.6, along with high K (keratometry) readings and thin corneal pachymetry, were classified as KCN or suspect KCN (SKCN). Pentacam HR images are self-explanatory and can easily diagnose KCN based on the abovementioned objective criteria as well as several other data points collected by the tomographer. A single examiner assessed the images and confirmed the diagnosis of KCN or SKCN.

The study adhered to the principles of the Declaration of Helsinki for research involving human subjects and received approval from the JHAH Local Institutional Review Board (IRB) office (approval number: 19-42).

Data and statistical analysis

The study involved screening 110 participants for KCN, with one participant excluded due to an inclusion criterion. Participant characteristics, including age, gender, nationality (Saudi/non-Saudi), systemic allergies/eczema presence, eye rubbing behavior, family KCN history, contact lens usage, and ocular measurements (e.g., BAD value, OD max k, minimum pachymetry) for both eyes, were measured.

Descriptive statistics, comprising means, standard deviations, counts, and percentages, summarized participant characteristics. Nonparametric comparisons, utilizing median values and interquartile ranges (IQR), were employed to assess ocular measurements across the KCN, normal, and SKCN groups. The gender distribution in each group was evaluated using chi-square tests. Significance was set at a p-value less than 0.05, and SPSS Statistics version 29.0 (IBM Corp. Released 2022. IBM SPSS Statistics for Windows, Version 29.0. Armonk, NY: IBM Corp.) was used to conduct the analysis.

## Results

The mean age of participants was 15.94 ± 2.88 years, with a majority being male (54.1%) and Saudi (76.10%). Systemic allergies, or eczema, were reported by 2.80% of participants, and eye-rubbing behavior was observed in 5.50% of participants. Approximately 21.10% had a family history of KCN, and 4.60% reported using contact lenses. KCN prevalence in the screening program was 2.75%, while the SKCN rate was 11.93% among total participants (Table 1).

**Table 1:** Descriptive statistics for participant characteristics BAD: Belin Ambrosio Display, SD: standard deviation, max k: maximum keratometry, SD: standard deviation

Variable	N	%
Age, m (SD)	15.94	2.88
Male	59	54.10%
Saudi	83	76.10%
Systemic allergies/eczema	3	2.80%
Eye rubbing	6	5.50%
Family history of KCN	23	21.10%
Contact lens user	5	4.60%
Right BAD value, m (SD)	0.87	0.86
Right OD max k, m (SD)	44.39	2.01
Right minimum pachy, m (SD)	554.55	38.83
Left BAD value, m (SD)	1.00	1.51
Left OS max k, m (SD)	44.76	3.54
Left minimum pachy, m (SD)	553.53	39.55

Descriptive statistics for ocular measurements revealed median OD max k values of 44.9, 44, and 45.6 for the KCN, normal, and SKCN groups, respectively (p=0.0012). Median values for minimum pachy were 492, 565, and 522 for the KCN, normal, and SKCN groups, respectively (p<0.0001). Similar patterns were observed for OS max k and left minimum pachy (both p<0.0001) (Table 2).

**Table 2:** Descriptive statistics and nonparametric comparisons for KCN KCN: keratoconus, SKCN: suspect keratoconus, IQR: interquartile range

Variable	KCN	Normal	SKCN	p-value
Right OD max k				
Median	44.9	44	45.6	0.0012
IQR	11.4	3.55	2.75	
Right minimum pachy				
Median	492	565	522	<0.0001>
IQR	48	68	70	
Left OS max k				
Median	57.1	44.1	45.6	0.0016
IQR	22.5	2.6	1.05	
Left minimum pachy 2				
Median	450	562	522	<0.0001>
IQR	76	39	47	

**Figure 1:**
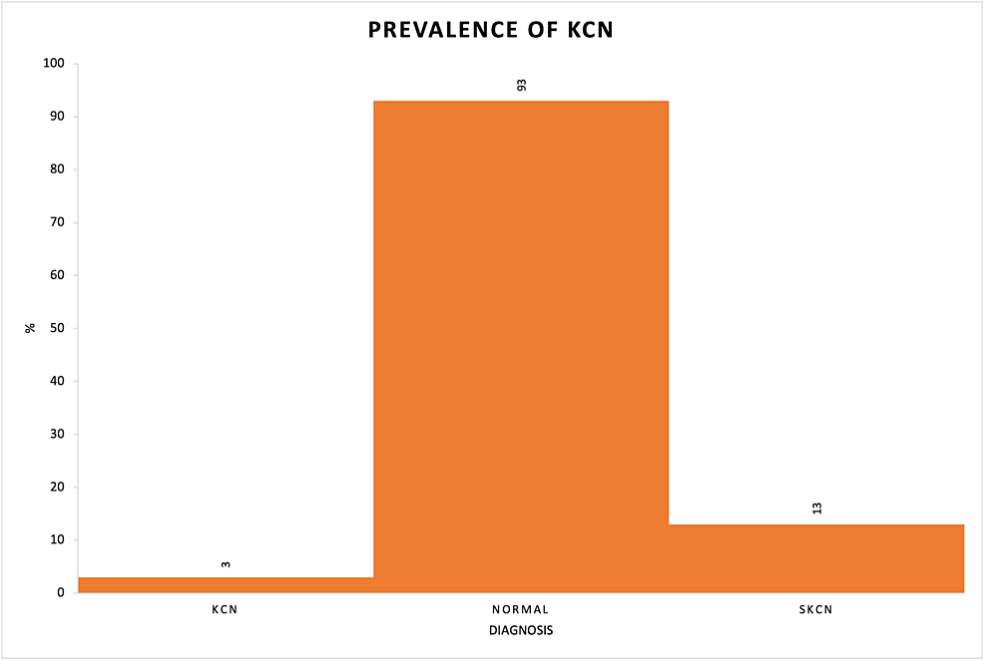
Distribution of KCN, normal, and SKCN participants KCN: keratoconus, SKCN: suspect keratoconus

Gender distribution analysis showed that in the KCN group, 33.33% were female and 66.67% were male. The normal group had 46.24% females and 53.76% males, while SKCN had 46.15% females and 53.85% males (p=0.909) (Table 3, Figure 1).

**Table 3:** Gender distribution and statistical comparisons for KCN KCN: keratoconus, SKCN: suspect keratoconus

Count Col % Row %	Female	Male	Total	p-value
KCN	1 2.00 33.33	2 3.39 66.67	3	0.909
Normal	43 86.00 46.24	50 84.75 53.76	93	
SKCN	6 12.00 46.15	7 11.86 53.85	13	
Total	50	59	109	

The prevalence of allergies, eye rubbing, family KCN history, and contact lens usage varied. Systemic allergies or eczema were reported by 2.80%, with none in the SKCN or KCN groups. Eye rubbing was reported by 5.50%, the highest in the KCN group (33.30%). A family history of KCN was found in 21.10%, with SKCN showing the highest prevalence at 30.80%. Contact lens usage was reported by 4.60%, with none in the SKCN or KCN groups (Table 4).

**Table 4:** Prevalence of risk factors among screened KCN: keratoconus, SKCN: suspect keratoconus

Variable	Normal		SKCN		KCN		Total	
Systemic allergies/eczema, n (%)	3	3.20%	0	0.00%	0	0.00%	3	2.80%
Eye rubbing, n (%)	5	5.40%	0	0.00%	1	33.30%	6	5.50%
Family history of KCN, n (%)	18	19.40%	4	30.80%	1	33.30%	23	21.10%
Contact lens user, n (%)	5	5.40%	0	0.00%	0	0.00%	5	4.60%

## Discussion

KCN, prevalent in the Middle Eastern region due to genetic and environmental factors, can be effectively treated with corneal collagen crosslinking, reducing further vision loss and ultimately the need for corneal transplantation [4-7]. The absence of early screening methods for diagnosis and treatment is a current challenge.

Historically, KCN diagnosis relied on placido-based topography or nomograms assessing risk [11]. As we advanced and corneal tomography evolved, our capability to diagnose KCN as well as SKCN with more accuracy increased. Initially, slit scanning devices, and now, Scheimpflug imaging and corneal tomography, have helped tremendously in the early diagnosis of the disease. Programs like Belin Ambrosio Enhanced Ectasia Display and ABCD staging available on Pentacam HR aid in identifying and staging KCN, allowing for timely intervention [12,13]. The development of tools for measuring corneal biomechanics has further aided in the early identification of KCN [14]. It has been demonstrated that corneal epithelial thickness mapping or other anterior segment coherence tomography devices are helpful in the diagnosis and tracking of disease [15]. We can see the changes occurring in the KCN cornea at a microscopic level thanks to corneal confocal microscopy.

With these tools, early disease diagnosis is possible, preventing severe consequences. Similar to amblyopia screening for kids, implementing widespread screening programs in middle and high schools could identify KCN in adolescents and initiate appropriate treatment. This initiative seeks data to support the development of these screening tools, particularly in regions where KCN is more prevalent.

There is significant variation in reported KCN incidence rates across studies. As mentioned in the Introduction, the 1986 study by Kennedy et al. in Minnesota reported an incidence of two per 100,0003, while a 2017 study in the Netherlands by Goddefrooij et al. found an incidence of 1:7500 or 265 cases per 100,00016. These figures were 5-10 times higher than previously reported values in population studies [3,16].

Most studies in the Middle East have indicated a significantly higher prevalence, ranging from 0.76% to 3.3% [4-7]. These elevated incidence rates underscore the need for robust population studies to validate the prevalence of KCN. Such studies are crucial for developing effective screening tools, particularly for adolescents in schools and colleges. Addressing this disparity will contribute to a better understanding and management of KCN in populations with varying prevalence rates.

**Figure 2:**
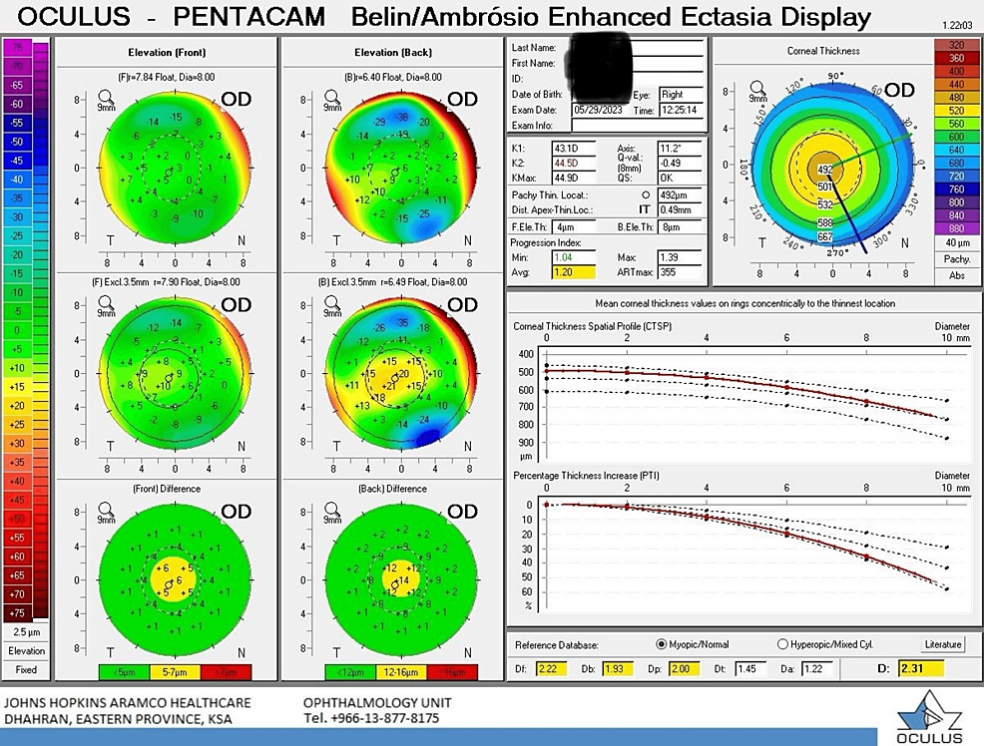
SKCN based on the BAD-D value of 2.31 BAD-D value: Belin Ambrosio Display D value, SKCN: suspect keratoconus

The utilization of the Belin Ambrosio Enhanced Ectasia Display D value as a criterion for identifying KCN suspects in the current study, especially those with a BAD-D value between 1.60 and 2.6 but without clinical signs or vision decline, aligns with findings from Hashemi et al.'s study on the top Pentacam HR indices. This emphasizes the relevance of the BAD-D value in diagnosing SKCN and definite KCN (Figure 2) [17]. The current study's data reveals a 2.75% incidence of KCN, which aligns with similar studies in the Middle Eastern region. While these findings are preliminary, anticipating higher numbers with a larger population screening is reasonable.

The observed strong correlation between family history and KCN, as well as the SKCN group, supports the notion of genetic causes as the likely etiology. However, there might be a small selection bias for this high correlation between family history and KCN, as people with a family history of KCN might be more eager to get screened for the disease. Additionally, our data reinforcing the role of eye rubbing in the disease's development is a significant finding. The high correlation of family history with these identified suspects underscores the importance of close follow-up and monitoring. As our study transitions to larger-scale investigations, it may reveal more cases of SKCN that require vigilant monitoring by ophthalmologists.

The current study is a useful pilot study, despite its limitations stemming from its restricted population from the Middle Eastern region and its inclusion of both Saudi and non-Saudi populations. Such regional studies will contribute to our understanding of the true prevalence of the disease in various nations and regions of the world and serve as a foundation for future, larger-scale research. Identifying patients who may be at risk for KCN may provide close observation for any signs of the condition progressing to KCN and prompt management, particularly in the younger population.

__PRESENT

## Conclusions

Multiple studies suggest that the Middle East has a higher prevalence of KCN than other parts of the world. To determine the true prevalence of the condition, larger-scale investigations must be designed. Large-scale research is necessary, and this may involve enrolling college and school students in KCN screening because the condition is curable if discovered early on. This would offer insightful information for upcoming initiatives and monitoring practices.
